# A Data-Based Framework for Identifying a Source Location of a Contaminant Spill in a River System with Random Measurement Errors

**DOI:** 10.3390/s19153378

**Published:** 2019-08-01

**Authors:** Jun Hyeong Kim, Mi Lim Lee, Chuljin Park

**Affiliations:** 1 Department of Industrial Engineering, Hanyang University, 222 Wangsimni-Ro, Seongdong gu, Seoul 04763, Korea; 2 College of Business Administration, Hongik University, 94, Wausan-ro, Mapo-gu, Seoul 04066, Korea

**Keywords:** source identification, sensor network, water quality monitoring, river system, statistical process control, random forest

## Abstract

This study addresses the problem of identifying the source location of a contaminant spill in a river system when a sensor network returns observations containing random measurement errors. To solve this problem, we suggest a new framework comprising three main steps: (i) spill detection, (ii) data preprocessing, and (iii) source identification. Specifically, we applied a statistical process control chart to detect a contaminant spill with measurement errors while keeping the false alarm rate at less than or equal to a user-specified value. After detecting a spill, we generated a nonlinear regression model to estimate a breakthrough curve of the observations and derive a characteristic vector of the estimated curve. Using the characteristic vector as an input, a random forest model was constructed with the sensor raising the first alarm. The model provides output values between 0 and 1 to represent the possibility of each candidate location being the true spill source. These possibility values allow users to identify strong candidate locations for the spill. The accuracy of our framework was tested on part of the Altamaha River system in Georgia, USA.

## 1. Introduction

Water is a crucial resource for both public health and ecological life. Since the amount of fresh water is decreasing at the same time that population, industrialization, and environmental pollution are increasing, the importance of water quality monitoring is attracting more attention. Based on improvements to real-time sensor and data analysis technologies that enable people to monitor water quality more effectively, the problem of identifying the source location of a contaminant spill has also been extensively studied by researchers. Most previous studies related to this problem have addressed two types of water systems: groundwater and rivers. Optimization algorithms, such as linear/nonlinear programming and meta-heuristics, have been commonly used to identify contaminant spill locations in groundwater systems, as shown by Aral and Guan [1], Aral et al. [2], Gorelick et al. [3], Singh and Datta [4], and Sun et al. [5]. Additionally, statistical methods (such as a backward probability model approach [6,7] and a geostatistical approach [8]) and machine learning techniques including artificial neural network models have been used by Singh and Datta [9], Singh et al. [10], and Srivastava and Singh [11] to address similar problems.

Due to the size and complexity of the problem, fewer studies have been conducted for rivers than for groundwater systems. Boano et al. [12] employed a geostatistical approach that generates previous information about a pollutant. Chen et al. [13] applied multivariate statistical methods to determine the spatial and temporal variations of water quality, then identified the contaminant source location. The backward probability method was also used by Ghane et al. [14] and Telci and Aral [15], while Lee et al. [16] recently provided a method based on random forest models to identify the source location of a contaminant spill in a river system. The random forest model is a famous classification model that consists of a set of tree-structured classifiers. It is known to have several advantages, such as computational efficiency, accuracy, and robustness, compared with other models. One may see Breiman [17] for an overview of the random forest model. For source identification in a river system, the random forest models provide values between 0 and 1, indicating the possibility that a candidate location is the true spill location, while the backward probability method provides only the rankings of candidate locations.

Many studies rely on hydrodynamic simulation models that provide contaminant concentration levels in a water system to develop and test their methods. It is difficult to apply these methods, however, because, in practice, the observed concentration levels often contain random measurement errors. As mentioned by Kim et al. [18], the results from a case considering observations with random measurement errors may be totally different compared with those from a case considering ideal observations generated by the simulation models without measurement errors (e.g., the concentration levels are exactly 0 when no spill event occurs). To obtain reliable results despite random measurement errors, one may need to control false alarm rates, which can be done by statistical process control (SPC) charts. A good review of SPC charts is provided by Montgomery [19]. Among the various SPC charts, we focus on the CUSUM chart developed by Kim et al. [20] due to the following advantages: (i) it enables users to derive a threshold value with a simple analytical method and (ii) it can deal with autocorrelated observations that follow a non-normal distribution.

This study, therefore, considers the problem of identifying a spill source location in a river system in the presence of random measurement errors. To the best of our knowledge, this is the first work considering random measurement errors in the source identification problem. To solve this problem, we suggest a new framework comprised of three main steps: (i) detecting a contaminant spill, (ii) preprocessing obtained contaminant spill data, and (iii) identifying the spill source location via random forest models. Specifically, we first use the CUSUM chart developed by Kim et al. [20] to detect a contaminant spill, where the false alarm rate is guaranteed to be less than or equal to a user-specified level. Then, we obtain a set of observation data including all information about the contaminant spill and use a robust locally-weighted regression model [3] to estimate the profile of the observations, called the breakthrough curve. Finally, using profile characteristics as inputs, we develop a classification method using random forest models to measure the possibility that each candidate spill location is truly the correct location of the contaminant source. Based on the possibilities, we can consistently identify strong candidate locations for the spill.

This paper is organized as follows. Section 2 describes the problem with notations and assumptions and provides a method for obtaining the observation data. In Section 3, we suggest a new framework to identify the source location of a contaminant spill in the presence of random measurement errors. Section 4 presents experimental results of a case study applied to a part of the Altamaha River system located in Georgia, USA. Concluding remarks follow in Section 5.

## 2. Background

### 2.1. Problem Description

We consider a river system including N  possible candidate locations for a contaminant spill. We index the locations by integers starting from 1 and call these integers location indices. In order to monitor the water quality and detect the spill event, K number of sensors are installed at a subset of the possible spill locations to report the concentration levels of the contaminant at each discretized time t.

Let D denote the index set of all possible locations, i.e., D={1,2,…,N}. For 2≤K≤N given, the location of the K sensors are represented by a vector $y=\left(y_{1},\ldots ,y_{K}\right)$, such that $y_{j}\in D$ for all j =1, …, K. (We assume $y_{1}<y_{2}<\ldots <y_{K}$ to avoid the repetition of representations). At each time t, an observation $X_{t}\left(y_{j}\right)$ is returned by the sensor installed at $y_{j}$ location, and the values from all K sensors at time t are represented by the observation vector:

(1) $$X_{t}\left(y\right)=\left[\begin{array}{c} X_{t}\left(y_{1}\right)\\ \vdots \\ X_{t}\left(y_{K}\right) \end{array}\right].$$

Since the true spill location is unknown and a contaminant spill can occur at any of the candidate locations, the possibility that location d∈D is the true source of the spill, P(d), can be evaluated. Note that 0≤P(d) ≤1 for all d∈D. The closer P(d) is to 1, the more likely that d is the source location of the spill. Let P denote a vector of P(d) as follows:

(2) $$P=\left[\begin{array}{c} P\left(1\right)\\ \vdots \\ P\left(N\right) \end{array}\right].$$

The problem considered in this paper is to construct a data-driven framework for the purpose of identifying the source location of a contaminant spill. We develop a model that calculates P based on the recent observation vectors, $X_{t-\omega +1}\left(y\right)$, ……, $X_{t}\left(y\right)$ with a pre-specified window length ω, when K and y are given.

To construct the data-driven framework and test its performance, preparation of a large dataset is required. In the next subsection, we briefly explain the data acquisition method by incorporating random measurement errors with values obtained from a hydrodynamics simulation.

### 2.2. Data Acquisition with Simulation

This paper supposes that an observation $X_{t}\left(y_{j}\right)$ may contain some measurement error for all t and j=1, …, K. In order to obtain realistic $X_{t}\left(y_{j}\right)$ values reported by the sensors with random measurement errors, we adopt the model from Kim et al. [18] as follows:

(3) $$X_{t}\left(y_{j}\right)=\xi_{t}\left(y_{j}\right)+\varepsilon_{\xi },$$

where $\xi_{t}\left(y_{j}\right)$ is the true concentration level of the contaminant and $\varepsilon_{\xi }$ is a random measurement error that depends on the value of $\xi_{t}\left(y_{j}\right)$.

For obtaining the realizations of $\xi_{t}\left(y_{j}\right)$ values, a popular simulation software package called the Storm Water Management Model (SWMM) provided by the United States Environmental Protection Agency, Durham, NC, USA, is used. The SWMM is developed by the United States Environmental Protection Agency to simulate hydrodynamics and contaminant transport in river systems under dynamic flow, including the various rainfall and watershed conditions described by Rossman [21]. The SWMM requires inputs of variable information related to the properties of random contaminant spills and rainfall events derived from historical data in addition to fixed information related to geologic/geometric properties and fundamental river system hydrodynamics. After executing the SWMM software using the inputs, we can obtain concentration levels from each candidate location at every inter-reporting time of the simulation clock.

As shown in Kim et al. [18], the measurement error $\varepsilon_{\xi }$ is quantified based on two perspectives: accuracy (i.e., the bias of the measurement error) and precision (i.e., the variability of the measurement errors). Let $\mu_{\xi }$ denote the level of accuracy and $\sigma_{\xi }$ denote the level of precision of the sensor at location $y_{j}$ at time t. For given values of $\mu_{\xi }$ and $\sigma_{\xi }$, $\varepsilon_{\xi }$ is assumed to be independent and identically distributed along with the Laplace distribution. Therefore, the probability density function of $\varepsilon_{\xi }$ is specified as follows:

(4) $$f{(\varepsilon }_{\xi }\;|\;\mu_{\xi },\;\sigma_{\xi })=\frac{1}{\sigma_{\xi }\sqrt{2}}\exp\left(-\frac{\sqrt{2}\;\left|\varepsilon_{\xi }-\mu_{\xi }\right|}{\sigma_{\xi }}\right).$$

## 3. Methods

### 3.1. Overall Description of the Proposed Framework

In this subsection, we provide notations and an overall description of our framework to identify the source location of a contaminant spill in the presence of random measurement errors. A list of notations needed to describe our framework is as follows:
tthe discretized time index at which each sensor returns an observation;$y^{*}$the location index of the sensor that raises the first alarm;$\tau_{a}^{*}$the point of time when the first alarm is on by the sensor at $y^{*};$
$\tau_{b}^{*}$the point of time when the first alarm is off by the sensor at $y^{*};$
$x_{th}$the concentration level reported by the sensor at $y^{*}$ at time $\tau_{a}^{*}$ (i.e., $X_{\tau_{a}^{*}}\left(y^{*}\right))$;ωa pre-specified window length for spill detection;ℓa lag parameter pre-designated by users to determine $\tau_{b}^{*}$;ψ(t)a nonlinear regression model for $X_{t}\left(y^{*}\right)$ in the time period [$\tau_{a}^{*},\;\tau_{b}^{*}];$
$B\left(y^{*}\right)$a vector representing the curvature characteristics of ψ(t);$D\left(y_{j}\right)$the set of candidate spill locations that can be identified only by the sensor at $y_{j};$ and$\phi \left(y_{j}\right)$the random forest model corresponding to the sensor at $y_{j}.$


**Figure 1:**
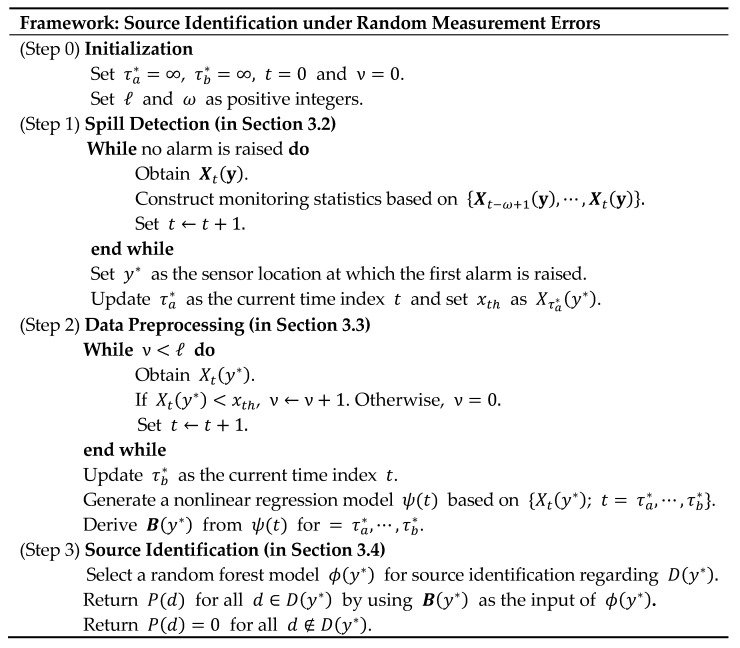
Overall description of the proposed source identification framework.

Figure 1 shows the overall description of our framework. Note that the framework consists of three main steps for (i) detecting a spill, (ii) preprocessing the set of obtained data, and (iii) identifying the source location with a classification model. Section 3.2, Section 3.3 and Section 3.4 provide details about each of the three steps, respectively.

### 3.2. Spill Detection

Detecting a contaminant source location under measurement errors is more complicated than detecting one without measurement errors. If there is no measurement error, the observed value is the same as the true contaminant concentration level and a sensor can simply raise an alarm when the observed value exceeds 0. In this case, an alarm is always a true alarm providing notification that a spill event has happened somewhere upstream. With measurement errors, however, nonzero values can be reported by sensors even when no spill event has occurred. In this case, users should be aware of the risk of false alarms and must carefully determine the threshold value for triggering an alarm. (A high threshold value increases the chance of missing a spill event, while a low threshold value subjects users to frequent false alarms.)

In order to detect a contaminant spill under random measurement errors, a statistical process control (SPC) chart with a carefully designed threshold can be used as a monitoring tool. The SPC chart was originally developed to detect a change in the mean of monitoring statistics while maintaining a targeted rate of false alarms. Among the various SPC charts, this paper introduces the CUSUM chart developed by Kim et al. [20] because it provides a simple analytical method for identifying a threshold value even for autocorrelated observations that follow a non-normal distribution. The target false alarm rate is proportional to a target in-control average run length (ARL), denoted by ρ, which represents the average number of observations until a false alarm is raised when the true mean of monitoring statistics is the same. Based on the target ρ given (i.e., the target false alarm rate given), Kim et al. [20] provide the threshold value H by solving the following equation:

(5) $$K\rho =\frac{1}{2\varsigma^{2}}\left\{\exp\left(\frac{2\varsigma \left(H+1.166\sigma_{0}\right)}{\sigma_{0}}\right)-1-\frac{2\varsigma \left(H+1.166\sigma_{0}\right)}{\sigma_{0}}\right\},$$

where ς represents a reference parameter and $\sigma_{0}$ is the known standard deviation of the random measurement errors. We use ς=0.1, which is recommended by Kim et al. [20]. We then construct our CUSUM statistics $S_{t}^{+}\left(y_{j}\right)$ for the sensor at location $y_{j}\;$ at time t with the monitoring window length ω as follows:
**Constructing CUSUM monitoring statistics**Set τ=t−ω and $S_{\tau -1}^{+}\left(y_{j}\right)\;=0$.**While**τ≤ t**do**  $S_{\tau }^{+}\left(y_{j}\right)\leftarrow \max\left(0,\;S_{\tau -1}^{+}\left(y_{j}\right)+X_{\tau }\left(y_{j}\right)-k\sigma_{0}\right)$.  τ←τ+1.**end while**

Once the threshold value H from Equation (5) and the monitoring statistic $S_{t}^{+}\left(y_{j}\right)$ are ready, an alarm is raised by a sensor at location $y_{j}\;$ at time t, when

(6) $$S_{t}^{+}\left(y_{j}\right)\geq H.$$

If the first alarm is raised by a sensor, we record the location of the sensor as $y^{*}$, the current time as $\tau_{a}^{*}$, and the concentration level $X_{\tau_{a}^{*}}\left(y^{*}\right)$ as $x_{th}$, before proceeding to the data preprocessing step.

### 3.3. Data Preprocessing

The main purpose of the data preprocessing step is to prepare refined input data for the random forest models to identify the source of the contaminant spill in the next step. This preparation is done by modeling the breakthrough curve of the observed concentration levels over time after the alarm [16] and deriving the characteristics of the curve.

**Figure 2:**
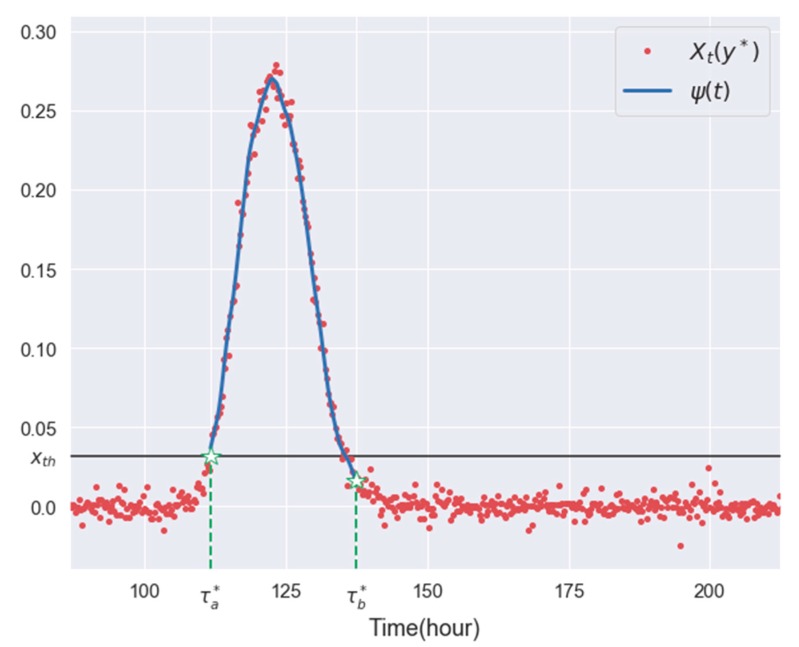
An example plot for $X_{t}\left(y^{*}\right),\tau_{a}^{*},\;\tau_{b}^{*},\;\mathrm{and}\;\psi \left(t\right)$.

To model the breakthrough curve that contains the most important information about the spill event, we must first define the start and end times of the curve. Because of random measurement errors, positive values of observations can be returned by a sensor even without any spill event. Thus, the time period considered for the curve should not be chosen simply as a period having positive values for returned observations. Instead, our framework suggests a heuristic approach to defining the start time of the curve as $\tau_{a}^{*}\;$ and the end time as

(7) $$\tau_{b}^{*}\equiv \min\;\{\;t\;|\;X_{p}\left(y^{*}\right)<x_{th}\;\mathrm{for}\;p=t-\ell ,\;\ldots ,\;t\;\mathrm{and}\;t\;>\tau_{a}^{*}\;\},$$

where ℓ is a lag parameter pre-designated by users. See Figure 2 for examples of $X_{t}\left(y^{*}\right)$ over time, $\tau_{a}^{*}$ and $\tau_{b}^{*}$.

Since random measurement error is similar to a noise factor, instead of using raw data $\left\{X_{t}\left(y^{*}\right);t=\tau_{a}\left(y^{*}\right),\;\cdots ,\;\tau_{b}\left(y^{*}\right)\right\}$ to represent the breakthrough curve, we used a nonlinear regression model to refine the curve. By reducing the impact of the random measurement errors with the regression model, a smoother and clearer breakthrough curve can be obtained. Among the various nonlinear regression models, we employ the robust locally-weighted regression model developed by Cleveland [22]. In this model, the independent variable is t and the dependent variable is $X_{t}\left(y^{*}\right)$ for $t=\tau_{a}^{*},\;\cdots ,\;\tau_{b}^{*}$. Let ψ(t) denote the concentration level estimated by the regression model at t. In Figure 2, the solid curve provides an example of the curve ψ(t) over time $t=\tau_{a}\left(y^{*}\right),\;\cdots ,\;\tau_{b}\left(y^{*}\right)$.

After obtaining the estimated breakthrough curve ψ(t), we need to derive its characteristics. We basically introduce two quantitative characteristics of ψ(t), denoted by $B_{1}\left(y^{*}\right)$ and $B_{2}\left(y^{*}\right)$, which represent the total area and the time-averaged area between the horizontal axis and the estimated breakthrough curve ψ(t), respectively [10]. Specifically, they are calculated as follows:

(8) $$B_{1}\left(y^{*}\right)=\int_{\tau_{a}^{*}}^{\;\tau_{b}^{*}}\psi \left(t\right)dt,$$

(9) $$B_{2}\left(y^{*}\right)=\frac{\int_{\tau_{a}^{*}}^{\;\tau_{b}^{*}}\psi \left(t\right)dt}{\left(\tau_{b}^{*}-\tau_{a}^{*}\right)}.$$

In addition to $B_{1}\left(y^{*}\right)$ and $B_{2}\left(y^{*}\right)$, we also consider the central statistical moment, standard deviation, skewness, and kurtosis of ψ(t)  as the main characteristics of the curve as described by Telci and Aral [15]. The first moment of the estimated breakthrough curve is denoted by $\mu \left(y^{*}\right)$ and calculated by

(10) $$\mu \left(y^{*}\right)=\frac{\int_{\tau_{a}^{*}}^{\;\tau_{b}^{*}}t\;\psi \left(t\right)dt}{B_{1}\left(y^{*}\right)}.$$

The estimated kth central moment of ψ(t), for k=2, 3, …, is denoted by $m_{\psi }^{k}\left(y^{*}\right)$ and calculated by

(11) $$m_{\psi }^{k}\left(y^{*}\right)=\frac{\int_{\tau_{a}^{*}}^{\;\tau_{b}^{*}}{\left(t-\mu \left(y^{*}\right)\right)}^{k}\;\psi \left(t\right)dt}{B_{1}\left(y^{*}\right)}.$$

The standard deviation, skewness, and kurtosis of the estimated curve, denoted by $B_{3}\left(y^{*}\right)$, $B_{4}\left(y^{*}\right)$, and $B_{5}\left(y^{*}\right)$ respectively, are calculated as follows:

(12) $$B_{3}\left(y^{*}\right)=\sqrt{m_{\psi }^{2}\left(y^{*}\right)},$$

(13) $$B_{4}\left(y^{*}\right)=\frac{m_{\psi }^{3}\left(y^{*}\right)}{{\left(B_{3}\left(y^{*}\right)\right)}^{3}},$$

(14) $$B_{5}\left(y^{*}\right)=\frac{m_{\psi }^{4}\left(y^{*}\right)}{{\left(B_{3}\left(y^{*}\right)\right)}^{4}}-3.$$

Based on Equations (8)–(14), the curvature characteristics of ψ(t)  are summarized into a five-dimensional vector as follows:

$$B\left(y^{*}\right)=\left(B_{1}\left(y^{*}\right),\;B_{2}\left(y^{*}\right),\;B_{3}\left(y^{*}\right),\;B_{4}\left(y^{*}\right),\;B_{5}\left(y^{*}\right)\right).$$

### 3.4. Source Identification

To identify the location of the contaminant source, we employed the random forest model shown in Lee et al. [16]. As inputs of the random forest model, Lee et al. [16] used not only characteristics of the breakthrough curves, but also time differences in alarms between any pair of sensors. Obtaining such relative time information, however, is often time-consuming and not helpful for improving source identification with random measurement errors. Therefore, we used only the characteristics of the estimated breakthrough curve $B\left(y^{*}\right)$ as inputs to our random forest models.

**Figure 3:**
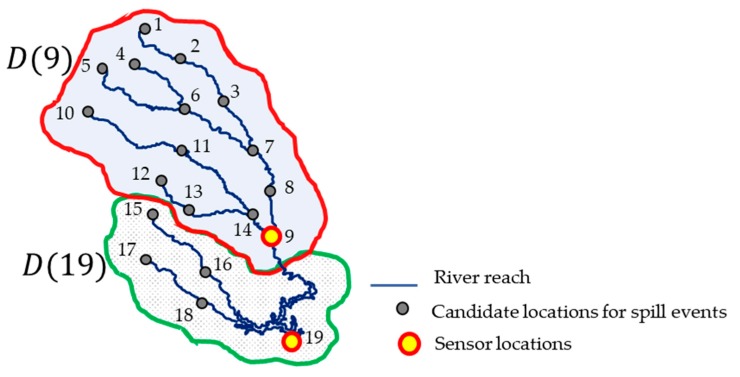
Examples of $D\left(y_{j}\right)$ when sensors are installed at locations 9 and 19.

In this study, we partitioned the whole river region into K sub-regions, each of which was monitored by a single sensor. Recall that $D\left(y_{j}\right)$ is defined as a set of candidate spill locations that can be identified only by the sensor located at $y_{j}$. For example, when N=19, K=2 and y=(9, 19), D(9)={1,2,⋯14} and D(19)={15, 16, 17, 18, 19} as in Figure 3. The sensor located at 9 raises the first alarm if a spill event occurs at any locations in D(9),  while the sensor located at 19 raises the first alarm if a spill occurs at any locations in D(19). In our framework, a random forest model was constructed corresponding to each $D\left(y_{j}\right)$ by using simulated training data that includes random measurement errors. Let $\phi \left(y_{j}\right)$ denote the random forest model regarding $D\left(y_{j}\right)$.

**Figure 4:**
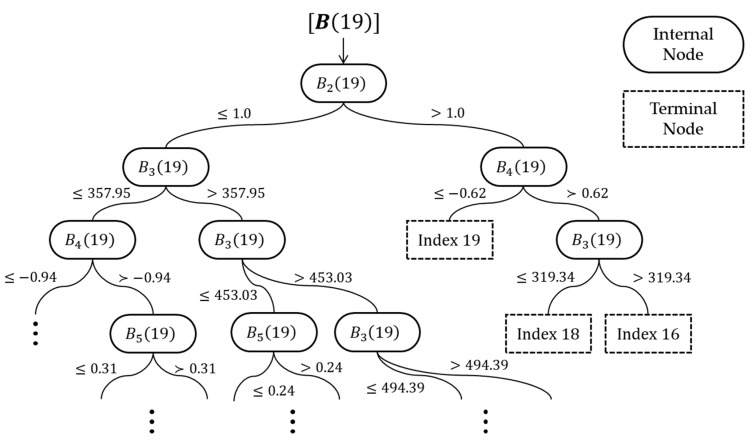
An example of part of a tree classifier.

**Figure 5:**
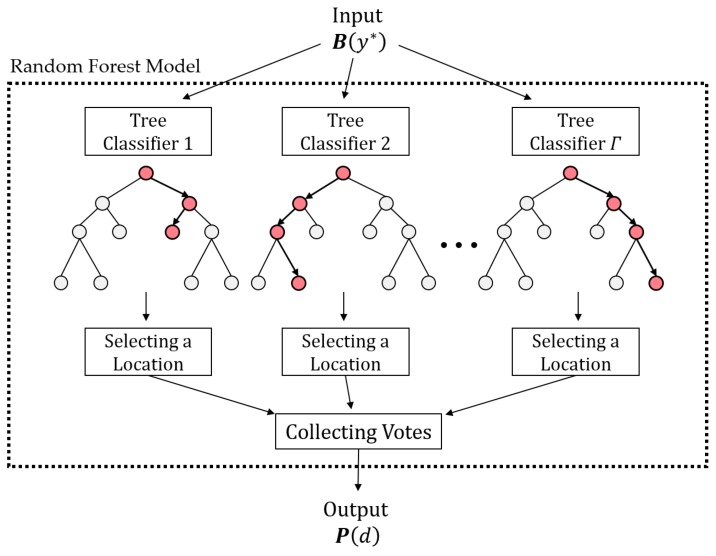
The overall structure of a random forest model for source identification.

The random forest model $\phi \left(y_{j}\right)\;$ consists of several tree classifiers. Suppose that we have Γ number of datasets sampled from the training data using a bootstrapping technique. Since a tree classifier is generated from each sample dataset, there are Γ number of tree classifiers in the random forest model. A tree classifier has internal nodes and terminal nodes. At each internal node, Λ number of input variables are randomly selected and linearly combined with coefficients. The linear combination of the selected input variables is checked if its value is above a certain threshold constant, and we move to the next node according to the result. To set the threshold constant and the coefficients of the linear combination at each internal node, a randomized node optimization algorithm is used [23]. Each terminal node represents one of the locations in $D\left(y_{j}\right)$ as the identified spill source location, and no additional decision or action is taken. Figure 4 shows a part of the tree classifier constructed for D(19) from Figure 3 as an example. Notably, different tree classifiers have different structures (e.g., the numbers of nodes and arcs) and logic, with different threshold constants and linear combinations for each internal node of the classifiers. If an input vector is given in the model, each tree classifier individually nominates one of the candidate locations as the identified contaminant source location. The random forest model $\phi \left(y_{j}\right)$ collects the votes from all tree classifiers and calculates P(d) for $d\in D\left(y_{j}\right)$ proportional to the number of votes from Γ. The model returns P(d)=0 for $d\in D^{c}\left(y_{j}\right)$. Figure 5 depicts the overall structure of a random forest model.

As remarked on by Lee et al. [16], Γ (i.e., the number of tree classifiers) and Λ (i.e., the number of input variables randomly selected at each internal node) affect the performance of the random forest model. As Γ increases, the error of the model gradually decreases and converges to a certain value; therefore, we use a sufficiently large value for Γ. Like Lee et al. [16], we selected Λ value as that with minimum error among the possible integers in [$0.5\sqrt{M},\;2\sqrt{M}]$ as well as [$0.5\left(log_{2}M+1\right)$, $2\left(log_{2}M+1\right)]$, where M is the number of variables in the input vector.

## 4. Case Study

### 4.1. Simulation Setup for the Targeted River System

In our case study, we applied our framework to monitor a part of the Altamaha River system in the state of Georgia, USA. The whole river system is approximately 760 km long and has over 36,260 km^2^ area with 60 reaches and 62 junctions. The fixed information for the SWMM, such as geometric, geologic, and hydrodynamic data, was obtained from the United States Geological Survey in the National Elevation Dataset, and spill and rainfall events are considered variable information for the SWMM. Spill starting time and intensity followed uniform distributions in [0, 10] days and [10, 1000] grams per liter, respectively. The rainfall pattern was randomly selected among five rain patterns for each of 10 predesignated sub-catchments for the river. See Park et al. [24] and Telci et al. [25] for more details about the river system and the corresponding SWMM model.

For each run, the SWMM monitors hydrodynamics and contaminant levels of spills and rainfalls for 40 days. The related quantitative values (e.g., concentration levels, flow rates, and the amounts of overflows) are reported every 15 min in the simulation clock at each candidate location. Each training and test dataset is obtained by adding random errors whose density function is provided in Equation (4) to the concentration levels returned by the SWMM.

### 4.2. Experimental Setup

**Figure 6:**
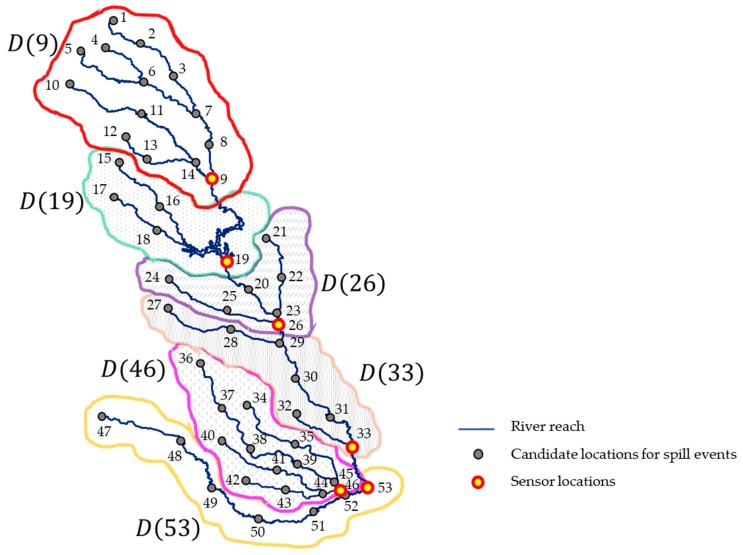
Targeted study area with six sensors.

We consider a part of the Altamaha River as a targeted study area, as seen in Figure 6. The study area includes 53 candidate spill locations (i.e., D={1,2,…,53}), which are marked as gray circles in Figure 6. Based on research from Lee et al. [16], we set the number of sensors K=6 and their locations, y=(9, 19, 26, 33, 46, 53). As mentioned in Section 3.4, the whole study area was divided into six sub-regions that were independently monitored by each sensor. The corresponding $D\left(y_{i}\right)$ values are represented in Figure 6.

For the random measurement errors, we consider two configurations to evaluate the impact of the errors: (i) low bias and low variability, denoted by (L, L), and (ii) high bias and high variability, denoted by (H, H). Specifically, we use $\mu_{\xi }=0.002\xi_{t}\left(y_{j}\right)$ and $\;\sigma_{\xi }=0.005+0.02\xi_{t}\left(y_{j}\right)$ for the (L, L) configuration and use $\mu_{\xi }=0.05\xi_{t}\left(y_{j}\right)$ and $\;\sigma_{\xi }=0.015+0.06\xi_{t}\left(y_{j}\right)$ for the (H, H) configuration as in Kim et al. [18]. For each of the random measurement error configurations, we searched the threshold value H of the CUSUM chart by using Equation (5) with ρ = 1000 (days) for each sensor (i.e., equivalent to the type I error, approximately $1.04\times 10^{-5}$). Under each configuration of measurement errors, we identified the control limit, H, for the CUSUM chart using Equation (5) when ρ=1000. Thus, we set H=0.228 for the (L, L) configuration and H=0.684 for the (H, H) configuration. The monitoring window length for the CUSUM chart, ω, is set to 10 days and ℓ is set to 7.

Table 1 summarizes information about random forest models constructed by evaluating $D\left(y_{j}\right)$s. For each sensor location $y_{j}$, the random forest model $\phi \left(y_{i}\right)$ is constructed (and trained) by using a $\left|D\left(y_{i}\right)\right|\times 500$ number of simulation datasets where |·| represents the cardinality of a set. As mentioned in Section 3.4, we checked various values of Γ (i.e., the number of tree classifiers in a random forest model) and Λ (i.e., the number of input variables randomly selected at each internal node), and finally selected Γ=500 and Λ=4 for all models. We used the “StatsModels” package to generate each robust, locally-weighted regression and the “scikit-learn” package to train each random forest model in Python version 0.21.2 (provided by the Python Software Foundation, Beaverton, OR, USA) with a personal computer (Intel Xeon E5-1650 CPU; RAM 64GB). The average time required to generate each random forest model was approximately 10.7, 3.1, 3.9, 3.8, 10.5, 5.2 s. Table 1 also provides the out-of-bag (OOB) errors for each random forest model, which was calculated by the ratio misclassified datasets to the total number of training datasets for $\phi \left(y_{i}\right)$.

### 4.3. Results

**Figure 7:**
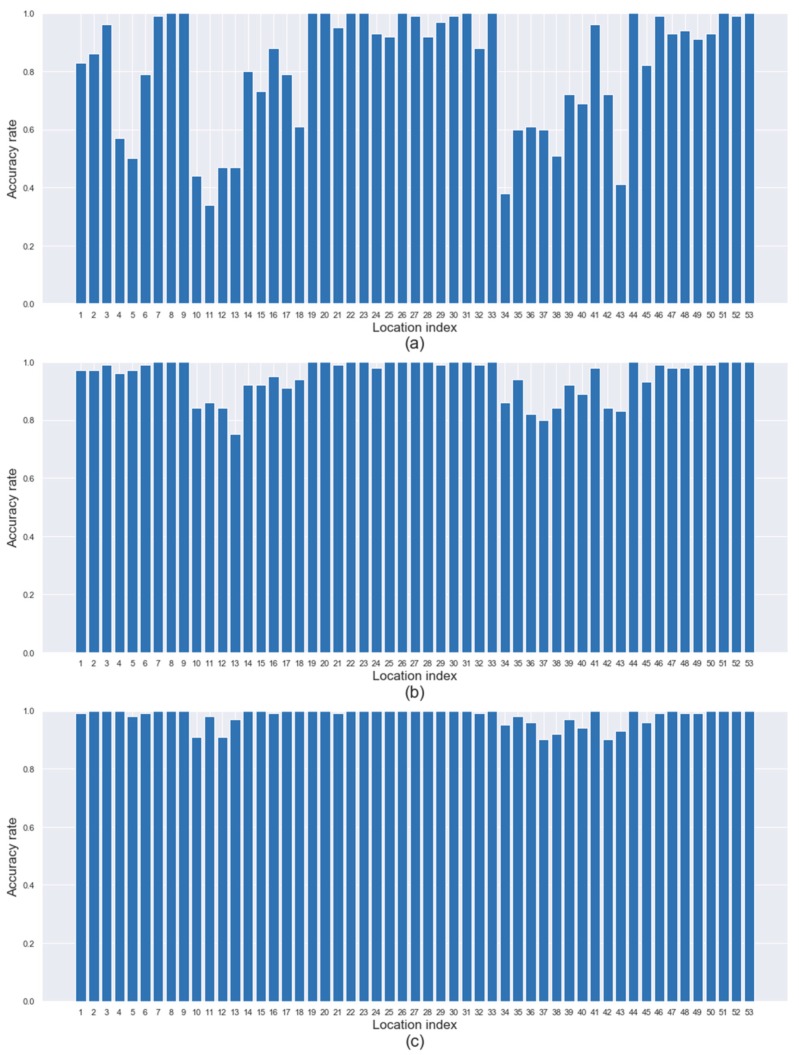
Identification accuracy regarding the top three locations under (L, L) configuration.

**Figure 8:**
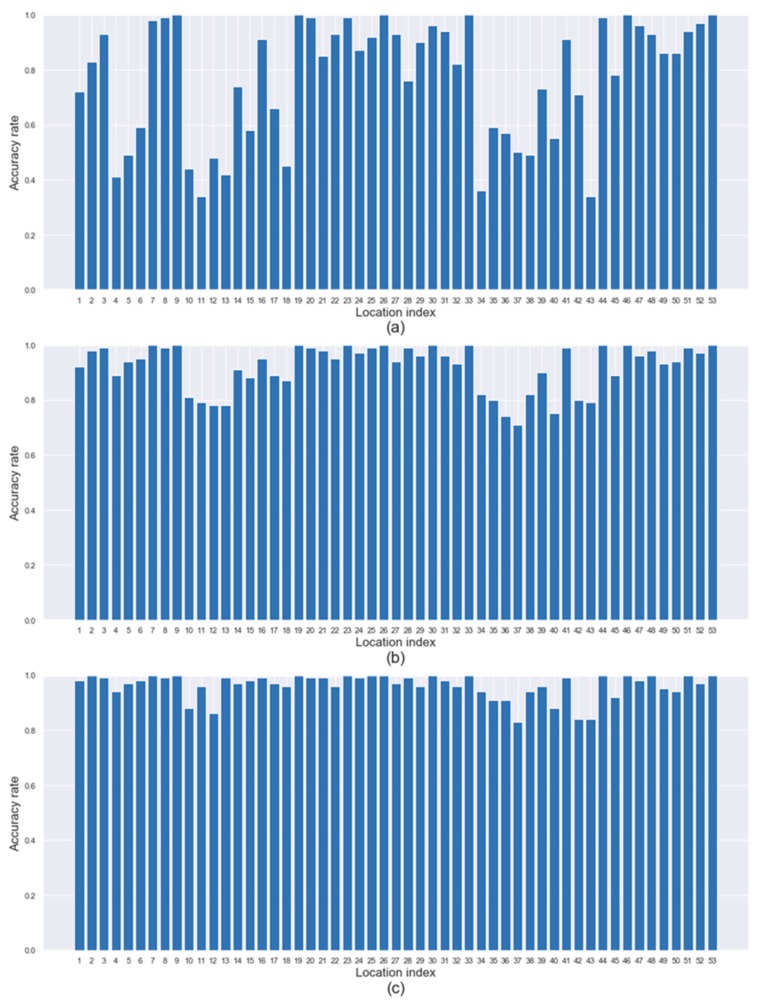
Identification accuracy regarding the top three locations under (H, H) configuration.

We ran our framework on 53×100 test datasets (i.e., 100 spills occurred at each of 53 candidate locations) to evaluate the performance of source identification. Figure 7 and Figure 8 show the identification accuracy rate (in *y*-axis) for the true spill location (in *x*-axis) under the (L, L) and (H, H) configurations, respectively. As the result of each test dataset, we can obtain a list of locations from the most promising to the least promising regarding the highest to lowest values of P(d) returned by our framework. Figure 7a presents the percentage of times (i.e., the rate) that the correct source location is the first place on the list. Figure 7b,c presents the percentage of times (i.e., the rate) that the correct source location is within the top two or three places on the list, respectively. Figure 7a shows that spills occurred at about one-fifth of the locations are identified with a relatively low accuracy rate of less than 60% while the spills that occurred at about half of the locations are identified easily with an accuracy rate of more than 90%. However, regarding Figure 7b,c, high accuracy rates of more than 90% are achieved for all 53 locations. In sum, no matter where the spill occurs, users can find the true spill location with more than 90% probability, if they visit the top three locations on the list. Figure 8 shows a similar pattern to Figure 7, but the identification accuracy rate in Figure 8 is slightly worse than that in Figure 7 because of higher bias and the variability of random measurement errors.

**Figure 9:**
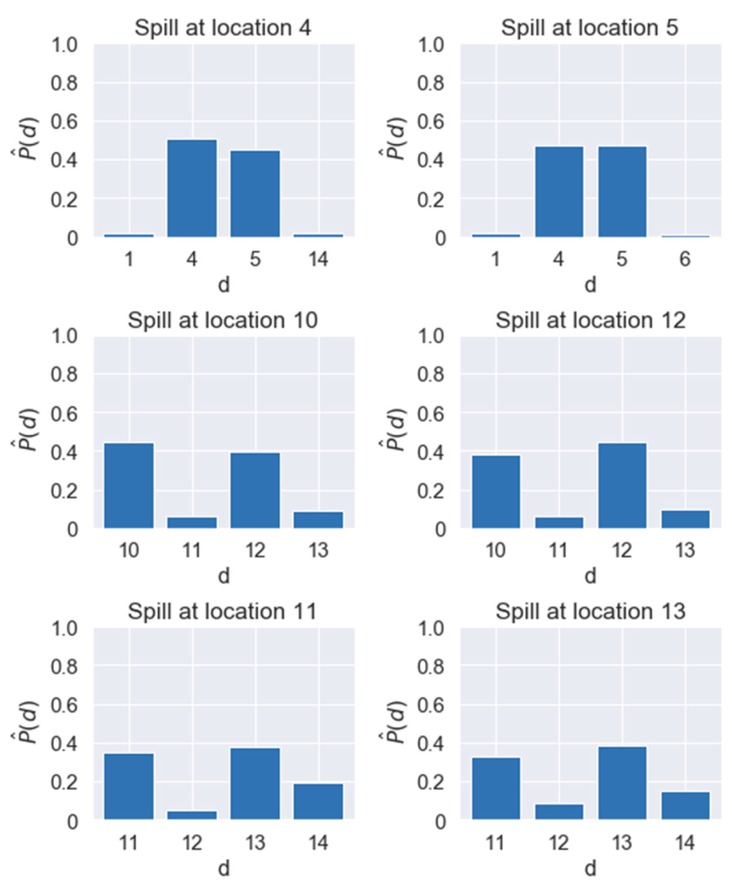
Examples of $\hat{P}\left(d\right)$ values for some related pairs of spill locations.

The random forest model is advantageous because it enables users to analyze the detailed possibility of the correct selection (e.g., P(d)). By comparing the obtained P(d) values, users can pick stronger candidates for the spill quickly and efficiently. For example, see Figure 9 representing the averaged P(d) values (denoted by $\hat{P}\left(d\right))$ at each location under the (L, L) configuration for some related pairs of locations. Let us consider the first pair (i.e., locations 4 and 5) in Figure 9, as an example. When a spill occurs at location 4 or 5, both locations 4 and 5 achieve significantly higher $\hat{P}\left(d\right)$ values than other locations. This implies that the spill at location 4 can often be confused with the spill at location 5. However, it implies that locations 4 and 5 can be recognized as promising spill locations when compared with other locations, regardless of where the true spill location is. (Note that similar analyses can be done for other pairs in Figure 9.) Thus, users can distinguish a group of stronger candidates based on gaps among P(d) values even when the true spill location is unknown, which is especially helpful in practical situations for finding unknown source locations under measurement errors.

## 5. Conclusions

This study proposed a new framework to identify the source location of a contaminant spill in a river system with random sensor measurement errors. In the framework, one may first detect a contaminant spill using the CUSUM chart while the type I error of detection is controlled. The framework generates a nonlinear regression model for observation data with random errors to estimate a breakthrough curve, and derives characteristics of the estimated breakthrough curve for use as inputs to random forest models. After selecting one random forest model corresponding to the sensor that detected the spill, values between 0 and 1 are evaluated for the possibility of each candidate location being the true contaminant source location. Based on the detailed values, users can judge how likely each location is to be the true spill location, and analyze the gaps among the values to distinguish the most promising candidate locations instead of just picking locations regardless of the possibility. The test results of applying our framework to the Altamaha River system in the USA show that our framework performs well on the identification of a spill source location, even with measurement errors. In the test results, the true spill location is listed within the top three promising locations with more than 90% probability in most of the cases considered.

Our framework was originally proposed to identify a contaminant spill source location in a river system. However, beyond water monitoring systems, the framework can be applied to other areas including traffic and transportation, manufacturing process monitoring, and telecommunications.

While conducting this study, we determined that the identification accuracy of the framework can be improved if the exact starting and ending times of the breakthrough curve caused by the spill event are known. Further research on improving the accuracy of these time indices is ongoing.

## Figures and Tables

**Table 1:** Random forest models with their OOB errors.

Sensor Location	Random Forest Model	Set of Candidate Spill Locations	% of OOB Error (L, L)	% of OOB Error (H, H)
9	ϕ(9)	D(9)={1, 2, 3, 4, 5, 6, 7, 8, 9, 10, 11, 12, 13, 14}	29.34	33.84
19	ϕ(19)	D(19)={15, 16, 17, 18, 19}	21.08	26.68
26	ϕ(26)	D(26)={20, 21, 22, 23, 24, 25, 26}	4.49	7.43
33	ϕ(33)	D(33)={27, 28, 29, 30, 31, 32, 33}	4.46	9.83
46	ϕ(46)	D(46)={34, 35, 36, 37, 38, 39, 40, 41, 42, 43, 44, 45, 46}	30.82	35.17
53	ϕ(53)	D(53)={47, 48, 49, 50, 51, 52, 53}	4.4	10.11
